# PHENOTYPIC STOCHASTICITY PROTECTS LYTIC BACTERIOPHAGE POPULATIONS FROM EXTINCTION DURING THE BACTERIAL STATIONARY PHASE

**DOI:** 10.1111/j.1558-5646.2012.01690.x

**Published:** 2012-06-11

**Authors:** Romain Gallet, Thomas Lenormand, Ing-Nang Wang

**Affiliations:** 1 Department of Biological Sciences, University at Albany1400 Washington Avenue, Albany, New York 12222; 2 CEFE–UMR 5175 1919 route de MendeF-34293 Montpellier, CEDEX 5, France

**Keywords:** Adsorption rate, heterogeneous phage subpopulation, phenotypic stochasticity, stationary phase, tail fiber

## Abstract

It is generally thought that the adsorption rate of a bacteriophage correlates positively with fitness, but this view neglects that most phages rely only on exponentially growing bacteria for productive infections. Thus, phages must cope with the environmental stochasticity that is their hosts’ physiological state. If lysogeny is one alternative, it is unclear how strictly lytic phages can survive the host stationary phase. Three scenarios may explain their maintenance: (1) pseudolysogeny, (2) diversified, or (3) conservative bet hedging. To better understand how a strictly lytic phage survives the stationary phase of its host, and how phage adsorption rate impacts this survival, we challenged two strictly lytic phage λ, differing in their adsorption rates, with stationary phase *Escherichia coli* cells. Our results showed that, pseudolysogeny was not responsible for phage survival and that, contrary to our expectation, high adsorption rate was not more detrimental during stationary phase than low adsorption rate. Interestingly, this last observation was due to the presence of the “residual fraction” (phages exhibiting extremely low adsorption rates), protecting phage populations from extinction. Whether this cryptic phenotypic variation is an adaptation (diversified bet hedging) or merely reflecting unavoidable defects during protein synthesis remains an open question.

It is argued that in an environment of homogeneous mixing, such as a liquid culture, a bacteriophage (phage) should evolve toward a high adsorption rate to maximize its fitness (Shao and Wang 2008). This conclusion neglects that, for many phages, not all hosts are equally worth infecting. While there are phages, such as T7, whose productive infection is independent of host physiological state (Schrader et al. 1997), most phages rely on the exponentially growing bacterial cells for productive infections (Adams 1959). Because natural bacterial populations are mostly in stationary phase or follow an alternation of exponential and stationary phases, it is conceivable that phages regularly have to cope with bacterial hosts in various physiological states, constituting uncertain environments.

Adaptation to environmental stochasticity (here, host physiological state) is a recurrent situation throughout the living world. For instance, in this context, phage adsorption to bacteria in stationary phase would be analogous, say, for a seed, to germinate in a bad year (Venable and Brown 1988): in both cases, no progeny are produced. Generally speaking, there are two main ways to adapt to fluctuating conditions: (1) evolution of phenotypic plasticity (e.g., developmental plasticity, inducible responses) or (2) evolution of a “robust“ phenotype that spreads the risk of adverse conditions—the so-called bet-hedging strategy (Lenormand et al. 2009). A bet-hedging strategy can be conservative (not involving phenotypic variation) or diversified (involving phenotypic variation, Philippi and Seger 1989).

The life cycle of temperate phages is a good example of how phages adopt the diversified bet-hedging strategy. After infecting a bacterial host, a temperate phage can go into the lytic program and starts producing virions or becomes a prophage by integrating its genome into the genome of its host (Hendrix et al. 1983 and references therein). More interestingly, the proportion of the infecting phage that goes through either the lytic or lysogenic route is dependent on the host physiological states, with more phages using the lysogenic route when the cells are in the stationary phase (Hendrix et al. 1983). By adopting such a strategy, similar to a form of dormancy, the phage population is protected from extinction during stationary phase.

By definition, strictly lytic phages cannot become prophages. Therefore, it is not clear how these viruses survive an adverse environment like the stationary phase. At least, three scenarios are possible. (1) Pseudolysogeny, a phenomenon similar to lysogeny, in which the infecting phage genome is not integrated into the bacterial genome but stays in the cytoplasm as an episome. This has been suggested as a potential mechanism for phage survival *in natura* (Ripp and Miller 1997; Williamson et al. 2001). (2) The production of virions with different adsorption rates by a single genotype (diversified bet hedging). Such phenomenon has already been observed in early phage literature: Schlesinger (1932) (translated in English by Stent 1965) reported the existence of a small subpopulation of phages with very low adsorption rates, referred to as the “residual fraction” and measured its proportion (0.3%). Those phages could be seen as a form of dormancy that allows phages to survive harsh seasons like stationary phase. (3) Selection of a phage genotype with an intermediate adsorption rate (conservative bet hedging). Like in most debate about the evolution of robustness, demonstrating whether these traits evolved to adapt to environmental stochasticity is difficult and often controversial. Indeed, robustness might have evolved as a side effect of other selective pressures or even as a result of maladaptation (Wagner 2005; Lenormand et al. 2009).

As strictly lytic phages can infect cells both in exponential or in stationary phase (Shao and Wang 2008; Gallet et al. 2009), yet cannot make either productive infections or lysogens when infecting stationary phase cells, we may expect high adsorption rates to be detrimental in stationary phase. While this hypothesis seems plausible, it has, to our knowledge, not been clearly evaluated. If true, the opposite selective pressures imposed on adsorption rate during exponential and stationary phase may indeed contribute to shape this trait and promote intermediate values (conservative or diversified bet hedging).

To better understand how strictly lytic phages survive during the stationary phase of their host, and how adsorption rate impacts this survival, we used two isogenic strains of the phage λ carrying the *cI*857 mutation (strictly lytic at 37°C), that differ in their adsorption rates. The fitness of each strain was measured in a series of three experiments. First, λ strains were exposed to *Escherichia coli* populations completing their entire growth cycle (exponential growth followed by a stationary phase). In a second experiment, we followed the viral populations during 24 h in the presence of *E. coli* in stationary phase. Finally, in a third experiment, we measured the adsorption rate of phages surviving stationary phase.

Our results show that in our experiment, pseudolysogeny was not responsible for survival and that, contrary to our expectations, high adsorption rate was not more detrimental during stationary phase than low adsorption rate. Interestingly, this last observation was due to the presence of the residual fraction, protecting the population from extinction. Whether this cryptic phenotypic variation is adaptive or merely reflects the occurrence of unavoidable phenotypic defects (e.g., protein synthesis errors) remains an open question.

## Materials and Methods

### BACTERIAL, PHAGE STRAINS, GROWTH MEDIA, AND PLATING CONDITIONS

Bacterial and phage strains used in this study are listed in Table 1. The main phenotypic difference among these phage strains is the adsorption rate, which is the result of carrying different side-tail fiber alleles (*stf^+^* or *stf^−^*). The adsorption rates for the Stf^+^ and Stf^−^ phages have been previously estimated with either the exponentially growing host (Shao and Wang 2008) or the stationary phase host (Gallet et al. 2009). In either host condition, the Stf^+^ phage has a much higher adsorption rate than the Stf^−^ phage. Two chromogenic markers, encoded by the *lacZα*^+^ (making blue lysis plaques) or *lacZα^−^* (making white lysis plaques) alleles, were also engineered into the phage genomes to discriminate between phage genotypes (Shao and Wang 2008).

**Table 1:** Bacterial and phage strains

Name	Relevant genotype	Reference
Bacterial strain		
IN24	Originally MG1655	*E. coli* Genetic Stock Center, #7740
IN28	Originally XL1 Blue	Stratagene (Gallet et al. 2009)
SYP045	MC4100(λ*cI857 R::LacZα^+^ J_WT_ stf^−^*)	(Shao and Wang 2008)
SYP046	MC4100(λ*cI857 R::LacZα^+^ J_WT_ stf^+^*)	(Shao and Wang 2008)
SYP049	MC4100(λ*cI857 R::LacZα^−^ J_WT_ stf^−^*)	(Shao and Wang 2008)
SYP056	MC4100(λ*cI857 R::LacZα^−^ J_WT_ stf^+^*)	(Shao and Wang 2008)
SYP132	MC4100(λ*cI857 J_WT_ stf^−^Δbor::kan*)	Unpublished data
Phage strain		
	All phages were obtained by thermally inducing the above lysogen strains	

The growth media of LB, TB, and H-top, and plating conditions have been described previously (Gallet et al. 2009). The kanamycin concentration in LB plates is 40 μg/mL.

### DYNAMICS OF PHAGE INFECTION

**Figure 1:**
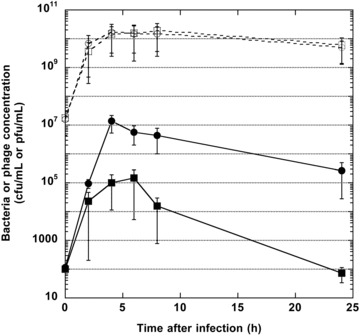
The population dynamics of phage strains and their corresponding bacterial hosts. λ phages with (Stf^+^, filled circles) and without (Stf^−^, filled squares) the side tail fibers were inoculated into cultures containing exponentially growing *E. coli* cells at time 0. Open symbols show the corresponding cell concentrations, vertical bars show the standard errors.

Exponentially grown *E. coli* cells at a concentration of ∼10^7^ cells/mL were infected with ∼10^2^ phages/mL in 10 mL LB medium in a 37°C waterbath shaker, mixing at 250 rpm. Culture samples, withdrawn at various time points, were used to estimate the bacteria and free phage concentrations. For the determination of the bacteria concentration, 50 μl of appropriately diluted sample was spotted directly on an LB agar plate. For the determination of the free phage concentration, the sample was first centrifuged at the maximum speed (∼14,000 ×*g*) for 1 min; the supernatant was then appropriately diluted and a 50 μl aliquot was spotted on a bacterial lawn that was freshly seeded with 100 μl of exponentially grown *E. coli* in 3 mL of H-top agar on an LB agar plate. The emerged colonies or plaques were counted after overnight incubation at 37°C. For each infection dynamics, three replicates were performed (Fig. 1).

### DYNAMICS OF PHAGE ADSORPTION TO STATIONARY PHASE *E. COLI* CELLS

The pattern of phage concentration decline in the presence of stationary phase host was determined by introducing approximately 10^10^ phages into a 10 mL, 24 h old *E. coli* culture grown in the LB medium at 37°C. Cell concentration after overnight incubation in a 37°C waterbath shaker shaking at 250 rpm is approximately 10^10^ cells/mL. The concentrations of both free phages and infective centers (free phage plus infected host cell [Adams 1959]) were estimated. Briefly, 100 μl of appropriately diluted sample were mixed with 100 μl of freshly grown *E. coli* XL1 Blue cells at room temperature. After 20 min of preadsorption, the mixture was then mixed with 3 mL of molten H-top agar with 14.3 mM Isopropyl β-D-1-thiogalactopyranoside (IPTG) and 0.06% X-gal and then poured onto a plate containing 40 mL of freshly prepared LB-agar. Plates were incubated overnight at 37°C before plaque counting. A total of four replicates were conducted. Because the host concentration is very high, great majority of the phages are expected to adsorb onto the cells. It is possible that the observed plaques may not be from the free phage fraction after the adsorption, but rather a result of contamination. To detect potential contamination, phages carrying the *lacZα^+^* marker were used. In this study, we did not observe any contamination.

### COMPARISON OF ADSORPTION PROFILES BETWEEN THE TOTAL PHAGE POPULATION AND THE T-2H POPULATION

Approximately 10^10^ *lacZα^+^* phage particles from the total population (i.e., original phage stock, composed of the main and the residual fraction) were incubated with 10 mL of overnight *E. coli* cells at 37°C for 2 h. The T-2h population corresponds to the free phage population remaining in solution after this 2 h exposure, and was collected after high-speed centrifugation. A final concentration of ∼10^3^ pfu/mL of *lacZα^−^* (resulting in white plaques) from the total phage population (the original phage stock) and ∼10^3^ pfu/mL of *lacZα^+^* (resulting in blue plaques) from the T-2h population were mixed together and then incubated with a 24 h old *E. coli* culture. Free phage concentrations were estimated before incubation, and then sampled from the culture 30 s, 7.5, 30, 60, and 120 min after incubation. The *lacZα* marker effect has been investigated in Gallet et al. (2009), but as phages do not reproduce in this experiment, we do not expect any marker effect.

### LYSOGENIZATION OF THE T-2H POPULATION

Phages carrying a kanamycin-resistance gene were obtained by thermal induction from lysogen SYP132 (Table 1) and used to obtain a T-2h population as described above. *Escherichia coli* cells were grown in TB medium plus 0.2% maltose for overnight at 37°C. Approximately 4 × 10^5^ phages from the T-2h population were incubated with 100 μl *E. coli* cells grown at room temperature for 2 h, instead of the usual 20 min, to facilitate adsorption. After adding 1 mL of LB medium to the bacteria-phage mixture and incubate at 30°C for 1 h, the culture was plated on kanamycin-containing LB plates and incubated at 30°C for overnight. With our T-2h population, only ∼3% phages successfully integrated into the bacterial genomes, resulting in kanamycin-resistant colonies.

### STATISTICAL ANALYSES

In our first experiment (Fig. 1), free phage concentration attains a maximum at the beginning of stationary phase (i.e., after ∼4 h), and then decreases because of adsorption. We analyze this decline using a survival analysis. We assume that a given phage has a constant adsorption rate α, which results in an exponential decay of the probability of remaining a free phage (note this probability *S*_α_(*t*) at time *t*). We have

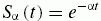
1

Further, we assume that all phages do not have the same adsorption rate. We assume that the adsorption rate among phages follows a gamma distribution with shape parameter *a* and scale parameter *b*

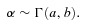
2

Integrating, the overall fraction of free phage in the population *S*(*t*) at time *t* is thus

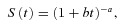
3
and the overall free phage population at time *t* is simply

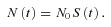
4

As phage population sizes are always estimated by serial dilution and plating, we assume that the number of plaque counted on a plate *n_t_* from a sample taken at time *t*, follows a Poisson distribution with a mean equal to *N*(*t*) divided by the dilution factor *d*

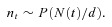
5

The likelihood of all the data are obtained by the product of the probability of observing each *n_t_* at all dilutions and time points. Parameters of the model (*N*_0_, *a*, *b*) can then be estimated by maximum likelihood. For the sake of a clearer interpretation, we reparameterized the model to fit the mean (α_*m*_=*ab*) and variance (α_*v*_=*ab*^2^) of adsorption rate in the population. Support limits were found within two units of log-likelihood corrected for overdispersion. Overdispersion was computed from the full model with each replicate fitted separately.

**Figure 2:**
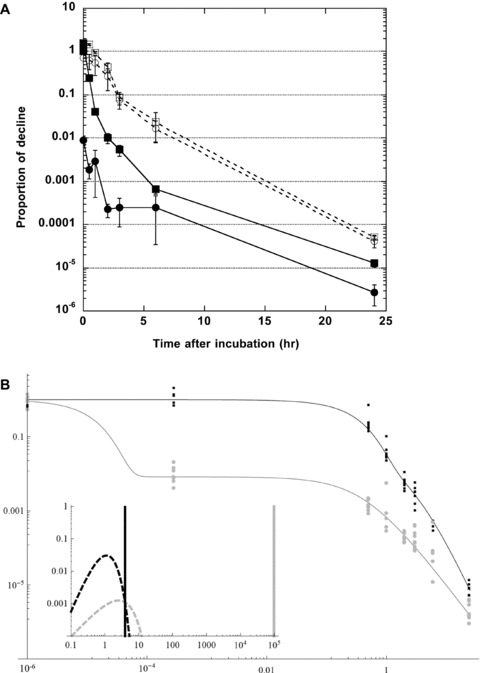
The dynamics of phage concentration decline when incubated with stationary phase cells. Approximately 10^10^ of either Stf^+^ (circles) or Stf^−^ (squares) phages were incubated with 10 mL of stationary phase *E. coli* cells (24 h old). Panel A: raw data. Filled symbols represent free phages and open symbols, infecting phages (total phages—free phages, i.e., phages currently infecting cells). Squares = stf^−^; circles = stf^+^. Vertical bars denote the standard errors. Panel B: raw data and model fit. On this figure, only free phages are shown, on a log–log plot to properly show the details of fit. Black squares = Stf^−^ data, gray circles = Stf^+^ data, lines represent statistical model fit. The inset shows the distribution of adsorption rates (black = stf^−^, gray = stf^+^) with adsorption rate as the *x*-axis, proportion of virus as the *y*-axis. Proportions of virus with a constant adsorption rate (main fraction) are represented by vertical bars, (95% stf^−^, 99% stf^+^) and proportion of phages with variable adsorption rates (residual fraction) are represented by probability densities (dashed curve).

**Figure 3:**
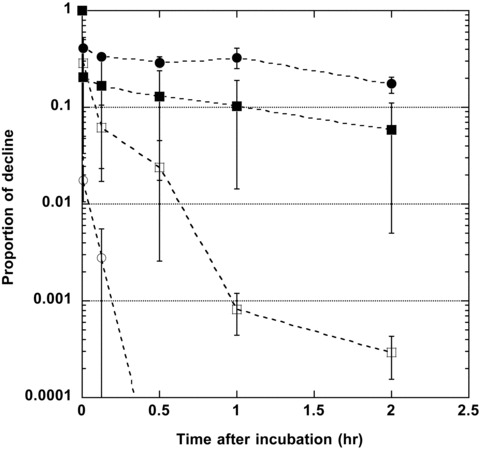
Adsorption dynamics of the original (open symbols) and the T-2h (closed symbols) phage populations. Approximately 10^3^ phages/mL of the Stf^+^ (circles) and Stf^−^ (squares) phages were used to initiate the study. The T-2h populations are the remaining free phages after 2 h incubation the stationary phase *E. coli* cells (see text for details). Vertical bars denote the standard errors.

In our second experiment (Fig. 2), phages were allowed to adsorb onto stationary phase cells for 24 h. We used the same analysis as above except that we assume that there were two populations of viruses: the main fraction with a constant and high adsorption rate and residual fraction with a gamma distribution of adsorption rate. *S*(*t*) in equation (4) was replaced by *S_mix_*(*t*):

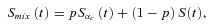
6
where α_*c*_ is the constant adsorption rate in the “main fraction” and *p* its proportion in the entire population. Finally, the same model used in experiment 1 was used to analyze data from our third experiment (Fig. 3). Computations were performed using Wolfram Mathematica 7.

## Results

### HIGH ADSORPTION RATE IS ADVANTAGEOUS THROUGHOUT THE HOST GROWTH CYCLE

To estimate the effect of adsorption rate on phage fitness during *E. coli*'s entire growth cycle (exponential and stationary phases) we used two isogenic λ strains that differ in their adsorption rates, as a consequence of the presence or absence of the side-tail fibers (Stf) (Hendrix and Duda 1992; Shao and Wang 2008). Our previous results showed that Stf^+^ phage has a much higher adsorption rate than the Stf^−^ phage, whether the host cells are in the exponential phase (Shao and Wang 2008) or the stationary phase (Gallet et al. 2009). Because the Stf^+^ phage has a high growth rate when the host is in the exponential phase, it can potentially deplete all host cells before the stationary phase is reached. To ensure that the phage strains would experience both the exponential and the stationary phases of the *E. coli* host, we used a high initial cell concentration of ∼10^7^ cells/mL and a low initial phage concentration of ∼10^2^ phages/mL for infection.

As shown in Figure 1, the concentrations of both the phages and the cells peaked at about 4 h after infection, reaching a maximum of ∼10^7^ and ∼10^5^ phages/mL for the Stf^+^ and Stf^−^ phages, respectively. This result is consistent with our previous finding that high adsorption rate is advantageous when in liquid culture with exponentially growing host cells (Shao and Wang 2008). While both phage concentrations started to decline 4 h after infection, there is no visible difference in the rates of decline (*F*_(2,8)_= 1.95, *P*= 0.2, see Table 2 for parameters estimations and support limits). Surprisingly, during the stationary phase, Stf^+^ phages did not incur the expected fitness cost compared to the Stf^−^ phage. Furthermore, even after 24 h, there were still free phages present in the cultures.

**Table 2:** Parameter estimates from statistical analyses performed in experiments 1, 2, and 3.

Exp.^a^		*P* (SL)^b^	α_*c*_ (SL)^c^	α_*m*_ (SL)^d^	α_*v*_ (SL)^e^
1	Stf^+^			0.50 (0.27– 0.82)	0.04 (0.01– 0.08)
	Stf^-^			0.37 (0.22– 0.63)	0.00 (0– 0.05)
2	Stf*^+^*	0.992 (0.987– 0.995)	10^5^ (2.5 × 10^4^–2 × 10^5^)	4.8 (3.6– 6.3)	10.7 (5.6–20.0)
	Stf^-^	0.946 (0.765– 0.963)	3.93 (3.40– 4.66)	1.54 (0.91–3.4)	0.65 (0.18–1.97)
3	Stf*^+^*			0.36 (0.27– 0.88)	0.0 (0.0 –1.89)
	Stf*^-^*			0.95 (0.68–1.41)	0.72 (0.11–2.85)
3^f^	Stf*^+^*			0.87	0.36
	Stf^-^			0.84	0.19

a Experiment.

b Proportion of phages with a constant adsorption rate α*_c_*.

c Constant adsorption rate estimate of phages belonging to the main fraction.

d Average adsorption rate of phages belonging to the residual fraction.

e Adsorption rate's variance of phages belonging to the residual fraction.

f α*_m_* and α*_v_* predicted from the model (eq. 7) with Δ*t*= 2*h* and parameters fitted in experiment 2.

### INFECTION OF STATIONARY PHASE CELLS IS DETRIMENTAL TO PHAGE FITNESS

To understand how lytic phages survive the stationary phase, we designed a second experiment by incubating a total of ∼10^10^ phages with 24 h old stationary phase cells for another 24 h. The fractions of free phages and infecting phages were estimated, respectively, by comparing the determined free phage concentrations and the calculated infecting phage concentrations (obtained by subtracting free phage concentrations from the infective center concentrations) to the input phage concentrations at time 0. As more phages are adsorbed onto the stationary phase cells, converting them from free phages to infecting phages, it is expected that the fraction of free phages would decrease with time. If a phage can survive inside a stationary phase cell as pseudolysogens, the infective center concentration should remain constant over the course of the experiment. On the other hand, if it is detrimental for a phage to infect a stationary phase cell, then we should expect to observe a decline in the concentration of the infecting phage.

Our results show an important decline of free phage as well as infective center concentrations (Fig. 2A). This later observation demonstrates that majority of phage λ cannot survive inside a stationary phase *E. coli* cell as pseudolysogens. At this point, it is not clear whether the genomes of infecting phages are destroyed by the stationary phase cells or the infected cells are killed without the production of phage progeny. In either case, it demonstrates that infection of the stationary phase cells does incur fitness cost to the infecting phages.

### THE ADSORPTION RATE IS HETEROGENEOUS WITHIN THE PHAGE POPULATION

Also shown in Figure 2A, with the first 30 min, only ∼0.2% of the Stf^+^ phages are still in the free-floating phase compared to ∼24% of the Stf^−^ phages, confirming that the Stf^+^ has a much higher adsorption rate than the Stf^−^ phage (see estimates in Table 2). However, the steep decline in free phage concentrations only occurred within the first hour of adsorption, followed by a slower decline at later times. To further explore this observation, we modeled the adsorption dynamics by assuming that the phage population consists of two subpopulations: one is composed of individuals with the same adsorption rate and the other of individuals with different adsorption rates.

Our statistical analysis provided an explanation for this slower decline. Phage populations were composed of two subpopulations. The first, that we call the main fraction represents ∼99% and ∼95% of the Stf^+^ and Stf^-^ populations, respectively. It is composed of individuals with a constant adsorption rate, certainly the wild-type phenotype. The second, the residual fraction (1% and 5% of Stf^+^ and Stf^-^ populations, respectively) is composed of phages with much lower and variable adsorption rates (see Fig. 2B inset for the fitted distribution of adsorption rates and Table 2 for estimates).

The existence of a small subpopulation composed of individuals with very low adsorption rates explains why there were still 1.4 × 10^−4^% and 1.3 × 10^−3^% of the initial phage populations of Stf^+^ and Stf^−^ phages respectively, in the free-floating phase after 24 h.

**Figure 4:**
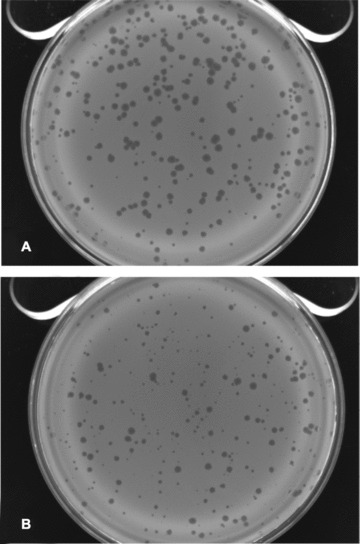
Plaque morphology of the original (A) and the T-2h (B) phage populations. Only Stf^−^ phage was plated.

Because we started with a high phage concentration for the adsorption dynamics, an alternative hypothesis to the existence of low adsorption phage subpopulations could be that most of the available binding sites on the cell surface for phage adsorption were fully occupied after initial incubation, thus leading to the slowed adsorption for the yet-unadsorbed phages. That is, if these later-time free phages were allowed to be adsorbed onto the freshly prepared stationary-phase cells, we should observe the same steep decline in phage concentrations as in the first 30 min shown in Figure 2. To test such a possibility, we collected the free phages after 2 h of preincubation with the stationary-phase cells for both the Stf^+^ and Stf^−^ phages (henceforth called the T-2h populations). The adsorption dynamics of the T-2h populations in the presence of freshly prepared stationary phase cells were then compared to the phage populations from the initial phage stocks, from which the T-2h populations were derived. As shown in Figure 3, with the starting phage concentrations of ∼10^3^ phages/mL, both T-2h populations showed a much slower decline than the stock Stf^+^ and Stf^−^ populations (likelihood ratio test, 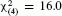, *P*-value = 0.003, see Table 2 for estimates and support limits). Furthermore, when plated on the agar plates, the proportion of Stf^−^ phages making small plaques is much higher with the T-2h population than with the initial phage stock population (Fig. 4). This observation is consistent with the extremely low adsorption rate exhibited by the T-2h phages. Indeed, adsorption rate is a determinant factor influencing plaque size. For instance, a high adsorption rate is detrimental in a viscous environment (Gallet et al. 2011). Thus, a phage with a low adsorption rate makes bigger plaques than a high adsorption phage. An extremely low adsorption is also detrimental, because the phage takes so much time to find a host that it will only complete few infectious cycles before the cells stop dividing (and the phage stop reproduce). The resulting plaque will be very small with a quite irregular shape, which was the case for the majority of individuals found in the T-2h populations (residual fraction).

Finally, we tested our statistical model by using survival curves from our second experiment (Fig. 2) to predict the adsorption rate of T-2h phages used in our third experiment (Fig. 3). Adsorption rate predictions given by the model were not significantly different from the adsorption rate measured in our third experiment (Table 2), demonstrating that our statistical model properly describe our empirical data.

### THE HETEROGENEOUS ADSORPTION RATE IS NOT HERITABLE

To determine whether the observed extremely slow-adsorbing phenotype associated with the T-2h population is due to mutation, we sampled individual phages in the T-2h population in the form of lysogens and plaques.

To facilitate lysogen selection, we used a λ strain that carries a kanamycin-resistance marker (see Table 1). Approximately 3% of the phages in T-2h population were integrated into the host genomes to form kanamycin-resistant lysogens. If the extremely slow-adsorbing phenotype is due to genetic mutation, then the second-generation T-2h population, obtained by thermal induction of the above lysogen population, should show a lower adsorption rate when compared to the original phage population. Otherwise, the adsorption rate would be the same as the original phage population.

**Figure 5:**
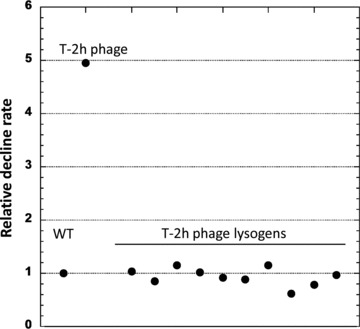
Decline rate of the wilt-type (WT) λ, T-2h λ, and 10 T-2h λ lysogens, relative to the WT.

We measured the adsorption rates of the second-generation T-2h populations derived from thermal induction of the kanamycin-resistant T-2h lysogen population (by collecting all colonies) as well as randomly picked individual T-2h lysogens. We found that the adsorption rates from the second T-2h populations are indistinguishable from the original phage population (Fig. 5).

We also extracted and plated the phages from 10 randomly picked small plaques like those shown in Figure 4B. If the small-plaque phenotype is due to genetic mutation(s), then the replated plaque size should be small as well. On the other hand, if the small-plaque phenotype is the result of phenotypic variation in adsorption rate with no genetic basis, then the replated plaque size should be the same as the parental size. We found that all replated plaques were large in size, as those shown in Figure 4A (data not shown).

These results demonstrated that the phenotype of extremely low adsorption rate assumed by the majority individuals of the T-2h population is not heritable.

## Discussion

In this study, we tested several hypotheses that could explain the maintenance of lytic phage populations during bacterial stationary phase. We first hypothesized that phages with intermediate adsorption rates should be favored when grown with bacteria during their entire growth cycle (exponential and stationary phases). This hypothesis was prompted by two observations: a high adsorption rate would be advantageous for phage fitness when the host cells are in the exponential phase (Shao and Wang 2008), but it would be disadvantageous when infecting cells in the stationary phase (see Fig. 2). Consequently, there should be an optimal adsorption rate that maximizes the phage fitness if the phage is constantly experiencing both the exponential and the stationary phases of its host. Despite empirical demonstration of the conditions necessary for a fitness trade-off, we did not observe the expected outcome. Instead, the high-adsorption phage (Stf^+^) maintained its advantage throughout the growth cycle of the bacterial host. Even though we did not include in our study other phage strains with intermediate adsorption rates, it is doubtful that any phage strain with an adsorption rate lower than that of the Stf^+^ strain would show a higher fitness.

We designed a second experiment to understand the processes occurring during stationary phase. First, we tested the hypothesis stipulating that phage populations could survive stationary phase as pseudolygens, a mechanism suggested in a previous study (Ripp and Miller 1997). Figure 2A shows a fast decline of the infecting phage population over only 24 h, demonstrating that λ cannot survive as a pseudolysogen in our experiment. This result could be seen as contradicting Ripp and Miller's conclusions (Ripp and Miller 1997). However, it is to be noted that the bacteriophage λ we used in our experiments is not naturally strictly lytic, but carries the thermosensitive mutation *cI*857 precluding lysogeny to occur at 37°C. Thus, *λ* has never been selected to survive as a pseudolysogen, which could explain why we do not confirm Ripp and Miller's results (Ripp and Miller 1997). Moreover, bacterial physiology in the natural environment of phage λ and in LB must be very different, which could be another explanation to this discrepancy. Second, we focused our attention on the free phage “survival” curves. We found that the reason why we did not observe an effect of adsorption rate on phage survival during stationary phase (Fig. 1) is because Stf^+^ and Stf^−^ phage populations were composed of two subpopulations, a main and a residual fraction. The main fraction represents the majority of the populations, and is composed of phages exhibiting a constant adsorption rate (see Table 2 and Fig. 2), while the residual fraction is heterogeneous, and composed of phages with variable (*α_v_* > 0) and much lower (*α_c_* > *α_m_*) adsorption rates. However, *α_c_* estimate in the Stf^+^ background should be taken with care. At such high bacterial concentration and phage intrinsic adsorption rate, it is hard to accurately measure adsorption rate. Snapshot measures are hard to perform (if possible), and measures on longer terms lead to a great overestimation because of the presence of the residual fraction. Phages were counted before and immediately (1 s) after inoculation of the bacterial population, but of course in practice, inoculation, sampling, and sample collection cannot be completed in 1 s only. Thus *α_c_* is probably greatly over or under estimated (see large support limits in Table 2). Besides *α_c_* estimate in the Stf^+^ background, our statistical analysis was good at estimating adsorption rates. For instance, we could predict the adsorption rate of phages sampled at time 2 h in experiment 3 with data from Figure 2 (see Table 2).

Our third experiment showed that the T-2h phages of the residual fraction exhibited an extremely low adsorption phenotype (Fig. 3) and did not “breed true” (Fig. 5), that is, the phenotype variation was not caused by genetic variation as observed previously by Garen (1954) and Sagik (1954). Because early studies of residual fraction (Delbrück 1942; Adams 1959) used phages other than λ, our results indicate that the heterogeneity in adsorption rate may be quite universal among tailed phages.

Most likely, the candidate responsible for the extremely low adsorption rate observed in the residual fraction is GpJ, the λ tail fiber protein that is involved in host recognition/adsorption and is common to both the Stf^+^ and Stf^−^ phages. We speculate that during the folding of the expressed GpJ, a minority of the protein alternatively folded (or misfolded) to assume a different conformation, possibly in the host recognition domain or the region(s) critical for trimer formation, thus resulting in extremely low adsorption rate.

The ability of phages to generate virions with different adsorption rate could have ecological consequences. Our results showed that in our experiments, free phage populations were protected from extinction by the presence of the residual fraction, despite the very high bacterial concentration. Thus, in the condition of our experiment, the presence of a residual fraction was adaptive (sensu, Reeve and Sherman 1993). However, whether this phenotypic variation occurs because of past selection of a diversifying bet-hedging strategy or because of the occurrence of unavoidable phenotypic defects (e.g., protein defect such as misfolding) remains an open question. Our results do not favor any of the diversifying bet hedging or the phenotypic defects hypotheses, over the other.

On the one hand, some studies have reported the evolution of diversifying bet-hedging strategy via individual phenotypic stochasticity. For instance, in isogenic cultures, persisters—cells stochastically entering in a dormant stage—allow bacterial populations to persist in the presence of “lethal” doses of antibiotics (see Lewis 2010 for a review). Some studies have also envisaged that protein defects could constitute a selective advantage upon environmental change (Whitehead et al. 2008; Drummond and Wilke 2009).

On the other hand, all processes are error prone, protein synthesis included (Drummond and Wilke 2009). In our case, GpJ protein synthesis errors may (1) have no consequence on adsorption rate, (2) cause defective phenotypes like low adsorption rates or, (3) be lethal. As these errors would not contribute to the residual fraction in cases (1) and (3), we can estimate that GpJ synthesis error rate is equal or greater than the observed residual fraction (between 0.1% and 0.01% of the total phage population, Fig. 2). In a recent review, Drummond and Wilke (2009) show that 0.1–0.01% of proteins, have misincorporated amino acids. If misfolding errors are also considered, the rate of protein synthesis error is predicted to be much higher than 0.1%. Thus, and as far as these figures applying to GpJ, the natural rate of protein synthesis error might be sufficient to explain the residual fraction.

There are several ways to further study these hypotheses. One would be to see if mutations that increase phenotypic stochasticity (similar to that of the *hipA* mutation in *E. coli* increasing the frequency of the persister phenotype (Moyed and Bertrand 1983) could also be identified in λ's genome.

Overall, our experiments showed that the residual fraction represented a very small fraction of the overall phage population (a result consistent with Schlesinger 1932 estimation) was composed of phages with variable adsorption rates (see our analysis) and resulted in phenotypic stochasticity (confirming; Garen 1954; Sagik 1954 conclusion that the typical “low adsorption rate” phenotype of phages from the residual fraction was not heritable). Furthermore, our study showed that, contrary to our expectations, a high adsorption rate was not detrimental to phage fitness during stationary phase, thanks to the presence of the residual fraction. This later result shows how the presence of this fraction can entirely change fitness measures of different phage genotypes and emphasizes that the stochastic phenotypic variation in a simple trait can play a significant role in shaping viral fitness. This finding was observed in a particular experimental setting; it remains to be determined if such phenomenon occurs frequently in nature, and if the residual fraction could be an adaptive response in phages to changing environments.
